# Molecular characteristics of the edge cells responsible for expansion of the chick embryo on the vitelline membrane

**DOI:** 10.1098/rsob.220147

**Published:** 2022-09-21

**Authors:** Hyung Chul Lee, Yara Fadaili, Claudio D. Stern

**Affiliations:** Department of Cell and Developmental Biology, University College London, Gower Street, London WC1E 6BT, UK

**Keywords:** cell polarity, epithelial sheet fusion, epithelial–mesenchymal transition (EMT), mesenchyme–epithelial transition (MET)

## Abstract

During early avian development, only a narrow band of cells (the edge cells, also called ‘margin of overgrowth’) at the rim of the embryo is responsible for blastoderm expansion by crawling over the vitelline membrane (VM) to cover the whole egg yolk in just 4 days (a process called epiboly). Surprisingly, this has not yet been studied in detail. Here we explore the edge cells of the chick embryo using *in situ* hybridization, immunohistochemistry and live imaging. Morphological and molecular properties reveal that the edge has a distinctive structure, being subdivided into sub-regions, including at least four distinct zones (which we name as leading, trailing, deep and stalk zones). This allows us to study reorganization of the edge region that accompanies reattachment of an explanted blastoderm to the VM. Immunohistochemistry uncovers distinct polarized cellular features resembling the process of collective cell migration described in other systems. Live imaging reveals dynamic lamellipodial and filopodial activity at the leading edge of the outermost cells. Our data provide evidence that edge cells are a distinct tissue. We propose that edge cells may be a useful model system for the study of wound healing and other closure events in epithelial cell sheets.

## Graphical abstract

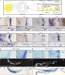

## 1. Introduction

**Figure 1.**
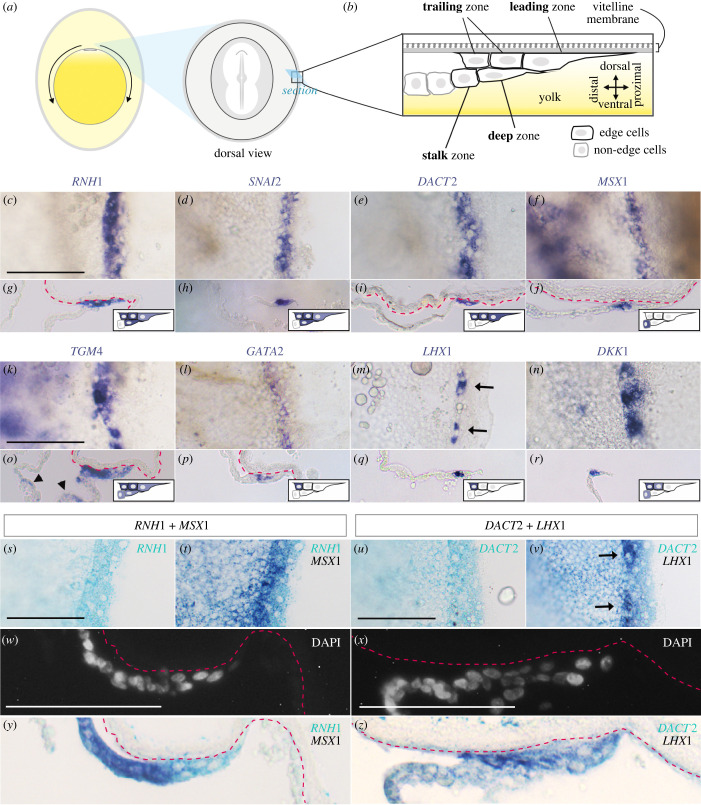
Expression patterns of selected markers for the edge region of the chick blastoderm. (*a*) Schematic diagrams of blastoderm expansion and the edge region at HH stage 6. Blastoderm expansion is driven by the edge cells and takes 4 days to cover the whole egg yolk. (*b*) Diagram of the edge region in section. Only the edge cells are attached to the VM. Edge cells and non-edge cells are outlined in black and grey, respectively. (*c–r*) The expression pattern of selected markers in the edge region, showing (*c–f*) and (*k–n*) dorsal view, and (*g*–*j*) and (*o*–*r*) section, with left to right showing proximal to distal. (*c*,*d*,*g*,*h*) *RNH1* and *SNAI2* are expressed in all edge cells. *DACT2* is expressed only in the upper edge cells including leading and trailing zones (*e*,*i*). (*f*,*j*) *MSX1* is exclusively expressed in the stalk zone. (*k*,*o*) *TGM4* is expressed strongly in the rear trailing zone but also weakly in other edge cells and some non-edge cells in a salt-and-pepper manner (arrowheads in (*o*)). (*l*,*p*,*m*,*q*) *GATA2* and *LHX1* are expressed exclusively expressed in the rearmost cell in the trailing zone (one-cell thick). *LHX1* is expressed heterogeneously along the edge region (arrows in (*m*)). (*n*,*r*) *DKK1* is strongly expressed in the rearmost cell in the trailing zone but also weakly in the distal cells of the trailing zone and in non-edge cells. (*s*–*z*) Double *in situ* hybridization of *RNH1*/*MSX1* (*s*,*t*,*w*,*y*) or *DACT2*/*LHX1* (*u*,*v*,*x*,*z*). (*s*–*v*) Expression patterns as seen in whole mount. (*w*–*z*) Expression in sections; nuclei stained with DAPI (*w*,*x*), *in situ* hybridization (*y*,*z*). The arrows in (*v*) indicate the expression of *LHX1*. Dashed magenta lines: ventral side of VM. Scale bars: 100 µm.

During the first few days of development of the chick embryo, the extraembryonic tissue (initially called the ‘area opaca’) at the periphery of the flat blastoderm gradually expands until, about 4 days' incubation after egg laying, it covers the entire surface of the spherical yolk mass. This represents more than a 200-fold increase in surface area and a rate of expansion of 200–550 µm h^−1^ [1] (figure 1*a*).This expansion is driven by specialized cells located at the extreme edge of the embryo, which are the only cells that adhere to the inner (yolk-facing) surface of the vitelline membrane (VM). This narrow ring of cells has been called ‘margin of overgrowth’ [2] and spans approximately 40–60 µm [1,3–5]. Several requirements need to be met for proper expansion of the blastoderm and normal embryo development. First, the attachment of this edge is required for blastoderm expansion and their outgoing movement generates and maintains tension in the embryo [4–6], which rises to a peak at around 20–24 h' incubation [1]. Second, tension in the acellular VM that constitutes the substrate for this expansion is also important for normal embryo development [4–6]. In addition, the orientation of both the embryo and the VM is important. For example, on an inverted VM, the embryo becomes abnormally strongly attached to the outer VM and does not expand, while an inverted blastoderm develops into a hollow vesicle probably by folding of the edge, allowing attachment of the dorsal side of the edge cells to the VM, on which they crawl to seal to form a sphere [5].

Cells at the edge of the embryo change their shape from initially cuboidal multiple layers to later a squamous and flattened monolayer [1,3]. The extreme edge region has larger cells with larger nuclei, while the immediately proximal region has densely packed cells [3]. Perhaps paradoxically given the rapid expansion of the circumference at this time, the extreme edge cells do not appear to proliferate [3,7]. It has been shown that proximal cells close to the edge contribute to the edge region; the same study also investigated some molecular markers expressed in this region and described specific expression of the intermediate filament protein, vimentin [7].

The edge cells have unique cellular morphologies that are related to their adhesiveness and migration. At their distal face, broad and flattened cell-processes adhere to the inner VM [8,9]. Incubation of embryos in small peptide inhibitors of adhesion to fibronectin causes transient detachment of the embryo from the VM and arrest of expansion for a short time, suggesting that fibronectin is involved in the adhesion of the embryo to the VM [10]. Chemical inhibition of microtubules also affects blastoderm expansion [11,12]. Apart from this, we know very little about the mechanisms of chick blastoderm expansion.

In this study, we revisit chick embryo expansion and study its molecular characteristics. We reveal molecular markers expressed in different sub-domains of the edge region. We show that when an embryo is removed from its membrane and then replaced onto it, reattachment is accompanied by an increase in the number of cells with edge cell properties (including molecular markers), suggesting that reattachment involves reorganization of the tissue. We use immunohistochemistry to explore various cellular properties including cell polarity, proliferation and proteins involved in cell adhesion—these are strongly reminiscent of similar properties described for cells undergoing collective cell migration in other systems. Finally, high-resolution live imaging reveals dynamic morphological changes of the most distal, leading-edge cells.

## 2. Results and discussion

### 2.1. Markers for the edge cells

No specific markers for edge cells have yet been described in any avian species. To find candidate genes that are specifically expressed in the edge region, we first examined whole-mount *in situ* hybridization data from previous studies (including [13] and data for a large number of genes from our laboratory). This revealed eight genes that are specifically expressed or highly enriched at the rim of the embryo: RNH1, SNAI2, DACT2, MSX1, TGM4, GATA2, LHX1 and DKK1 (table 1). To investigate these in more detail, we performed *in situ* hybridization on embryos incubated for 24 h (approximately HH stage 5–7 embryos [14]. In dorsal view, all the genes showed localized expression in the outermost 2–3 cells all around the periphery of the embryo (figure 1*c–f* and *k–n*); in the case of *MSX1* and *TGM4*, expression was also seen in non-edge proximal cells (figure 1*k,f*). Histological sections of these embryos revealed distinctive expression patterns of the markers for cells located at slightly different positions within the edge region. This allows classification of four distinct regions, which we name **leading** (at the leading/free edge, and adjacent to the VM), **trailing** (also adjacent to the VM but just behind the leading cells), **deep** (underlying the trailing zone, towards the yolk) and **stalk** zones (also deep, towards the yolk but further removed from the leading edge) (figure 1*b*). The cells in all four zones have flattened morphology and much larger nuclei and cell size compared to other area opaca cells more distant from the edge (non-edge cells), but the four zones differ from each other. Extensive membrane ruffles are seen particularly in the leading zone, extending mostly towards the direction of migration (figure 1*b*). Cells in the stalk zone provide a connection between the edge region and non-edge cells (figure 1*b*).

**Table 1.** List of marker genes for the edge region.

gene	NCBI gene ID	full name	marker of
RNH1	423111	ribonuclease/angiogenin inhibitor 1	leading/trailing/deep
SNAI2	432368	snail family transcriptional repressor 2	leading/trailing/deep
DACT2	421561	dishevelled binding antagonist of beta catenin 2	leading/trailing
MSX1	396484	msh homeobox 1	stalk
TGM4	420706	transglutaminase 4	leading/trailing/deep/stalk
GATA2	416018	GATA binding protein 2	trailing
LHX1	396381	LIM homeobox 1	trailing
DKK1 (DKK4)	374156	dickkopf WNT signalling pathway inhibitor 4	trailing/stalk

This classification reveals quite specific expression patterns for the eight markers (table 2). Figure 1*c–r* shows representative images in dorsal and sectioned view. *RNH1* and *SNAI2* are expressed in all edge cells but more weakly in the leading zone (figure 1*c,d,g,h*). *DACT2* is expressed specifically in upper cells (leading and trailing zones, adjacent to the VM) but not in cells in the deep zone (figure 1*e,i*). *MSX1* is specifically expressed in the stalk zone (as well as in neighbouring non-edge cells), but absent in all the other edge sub-regions (figure 1*f,j*). *TGM4* is expressed strongly in the trailing zone but weakly in the other edge zones and non-edge cells (figure 1*k,o*). Both *GATA2* and *LHX1* are expressed exclusively in the trailing zone furthest from the leading edge (1 cell thick) (figure 1*l,m,p,q*), while the latter showed mosaic (heterogeneous) expression in the trailing zone furthest from the leading edge (figure 1*m*). *DKK1* is expressed in the trailing zone and also occasionally in stalk zone (figure 1*n,r*). To confirm these results, we used double *in situ* hybridization to visualize the expression of either *RNH1* with *MSX1,* or of *DACT* with *LHX1,* in the same embryos. *RNH1* and *MSX1* are expressed in separate regions: *RNH1* in all edge cells except in the stalk zone, while *MSX1* is expressed exclusively in the stalk zone (figure 1*s,t,w,y*). The expression of *DACT2* and *LHX1* overlaps, the latter being heterogeneous (mosaic) in the rearmost cells of the trailing zone (figure 1*u,v,x,z*).

**Table 2.** Details of expression of markers in the edge region. Here, the rear zone includes the trailing zone and the deep zone. Numbers: (number of sections with expression) / (total number of sections). n/e: not expressed.

	leading zone	rear zone	sub-regions of rear zone		stalk zone
			trailing zone	deep zone	
*RNH1*	33/33	33/33	33/33	29/33	0/33
*SNAI2*	10/14	13/14	14/14	13/14	0/33
*DACT2*	20/30	30/30	30/30	5/30	0/33
*MSX1*	0/21	0/21	n/e	n/e	21/21
*TGM4*	11/25	23/25	23/25	15/25	13/25
*GATA2*	0/19	19/19	19/19	0/19	0/19
*LHX1*	0/20	20/20	20/20	0/20	0/20
*DKK1*	0/26	19/26	14/26	7/26	10/26

Next, to investigate if these patterns of expression are maintained throughout the process of expansion, two more stages, 12 h (approx. HH stage 2–3, an early stage of expansion) and 3 days (approx. HH stage 19, when the edge has passed the equator and is now close to the South pole of the yolk), were investigated for expression of *RNH1*, *SNAI2*, *DACT2* and *MSX1*. At 12 h, three of the markers (*RNH1*, *SNAI2* and *DACT2*) are expressed strongly in edge cells, while *MSX1* is expressed in the stalk zone and non-edge cells (electronic supplementary material, figure S1A–L), which is comparable to 24 h embryos in figure 1. Unlike 24 h embryos, expression of *RNH1*, *SNAI2* and *DACT2* in non-edge cells is also observed (electronic supplementary material, figure S1I–J). Also, the edge region as a whole at 12 h contains fewer cells in thickness (electronic supplementary material, figure S1E–L) compared to 24 h (figure 1). On the other hand, at 3 days (electronic supplementary material, figure S1M), the edge shows fairly regular, marked infoldings, suggesting that this may be a mechanism for reducing the circumference of the blastoderm edge as embryo expansion reaches its end (electronic supplementary material, figure S1N). All four markers are specifically expressed in the edge cells, but *MSX1* is only expressed in the stalk (electronic supplementary material, figure S1O–Z). At 3 days of incubation, the edge has thickened, involving more cell layers, in comparison with a very thin, one-cell layer of non-edge cells (electronic supplementary material, figure S1S–Z). This could be an additional mechanism contributing to reducing the circumference of the edge as expansion around the yolk nears completion. These results reveal that, throughout embryo expansion, edge cells maintain expression of the markers while they change their cellular composition, suggesting that some of these genes may have a role in the migration of the edge cells.

Together, our data reveal that the edge region of the embryo can be subdivided into at least four regions using different markers (table 1), implying different biological properties of edge cells in each zone and defining a distinctive structure for the edge region as a whole.

### 2.2. Reorganization of the edge region during reattachment process

**Figure 2.**
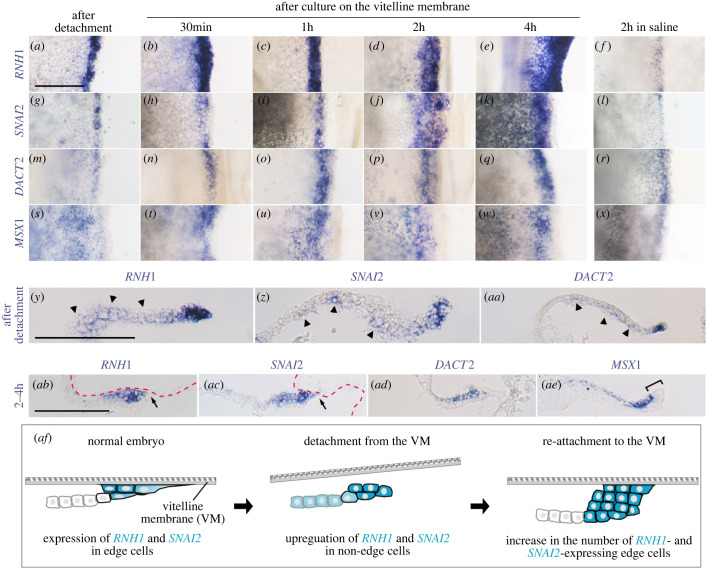
Reorganization of the edge during reattachment. Embryos at HH stage 6 were detached from VM and then cultured by replacing them onto the VM for different time periods before assessing the expression of edge cell markers *RNH1*, *SNAI2*, *DACT2* and *MSX1*. As controls, (*f*,*l*,*r*,*x*) show the expression of the same four genes in embryos incubated in suspension in saline for 2 h. After detachment, the former three makers are induced weakly in non-edge cells proximal to the edge region (arrowheads in (*y*–*aa*)). During reattachment, all genes show a gradual increase in the width of the expression domain, especially from 2 h after culture (d,*j*,*p*,*v*). During reattachment, there is an increase in cell layers in the edge, including expression of *RNH1*, *SNAI2* and *DACT2* (*ab*–*ad*). From 2 h, the front-most edge cells flatten (arrows in (*ab*,*ac*)). *DACT2* expression is limited to upper edge cells (*ad*). The expression of *MSX1* is absent from the front edge cells (bracket, (*ae*)). Dashed magenta lines: ventral side of VM. Black arrows: attachment points of the embryo to the VM. White arrows, flattening of the cell in the leading zone. Scale bars: 100 µm. (*af*) Schematic summary of cellular changes that accompany the reattachment process.

Next, we investigated the behaviour of the edge region that accompanies attachment to the VM, using the newly found markers. Embryos were detached from the VM, kept in saline for 30 min before replacing them on the VM and incubation for different periods of time, followed by *in situ* hybridization to check the expression of edge cell markers (figure 2). *RNH1* and *SNAI2* were selected as they are expressed in all four zones, while *DACT2* and *MSX1* were selected because they mark upper edge cells and cells in the stalk zone, respectively.

Several distinct features were observed during the reattachment process. First, three of the four markers (*RNH1*, *SNAI2* and *DACT2*) are upregulated in non-edge cells just proximal to the edge soon (30 min) after detachment from the VM (figure 2*a,g,m,y*–*aa*). Second, all four genes gradually expand their expression domains during culture, from a width of 2–3 cells before culture to a maximum of 10 cell diameters from the edge as seen in dorsal view, and 4–6 cells in depth as shown by sections after 2 h (figure 2*a*–*x*, *ab*–*ae*). The greatest rate of expansion occurs by 2 h (figure 2*d,j,p,v*). This expansion of the expression domains depends on attachment to the VM, as embryos kept in saline for 2 h showed only weak expression and no expansion of the expression domains (figure 2*f,l,r,x*). Finally, during reattachment, the marker genes maintain their expression pattern (spatial expression in different zones) regardless of the extra thickness due to additional cell layers (figure 2*ab*–*ae*). For example, *RNH1* and *SNAI2*, which are initially expressed in all four zones, are still expressed throughout the edge after reattachment (figure 2*ab*,*ac*). *DACT2* maintains its expression, limited to the upper cell layer (figure 2*ad*), and *MSX1* is expressed in rear cells (like the stalk zone) but is absent from distal cells (figure 2*ae*).

The cell morphology, including nuclear shape, of edge cells quickly becomes rounded after detachment (figure 2*y*–*aa*) and does not fully recover even several hours after reattachment (figure 2*ab*–*ae*). Some of the leading cells start to flatten again at 1 h and most of them have done so 2 h after replacing the embryo on the VM (figure 2*ab*–*ae*). Figure 2*af* presents a schematic summary of the cellular changes seen during the reattachment process.

Additionally, the increased adhesion to the VM was also apparent by the embryo retaining its association with the membrane even after the harsh *in situ* hybridization process from 2 h onwards. Attachment of non-edge cells was frequently observed at multiple points (electronic supplementary material, figure S2A,C). Occasionally, the edge folded back on itself, and the ventral face of the edge region became attached to the VM (electronic supplementary material, figure S2B).

Taken together, these results reveal that during reattachment, the edge region of the embryo increases the number of cells with edge cell identity, recruiting neighbouring non-edge cells and thus becoming more closely associated with the VM.

### 2.3. Cellular properties of edge cells: cell polarity, extracellular matrix, the cytoskeleton and proliferation

**Figure 3.**
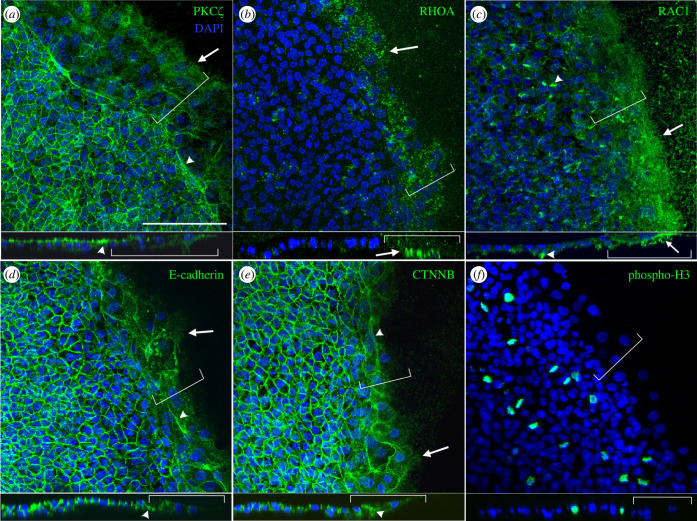
The expression of markers of cell polarity (PKC*ζ*, RHOA and RAC1), cell-to-cell adhesion (E-cadherin and CTNNB), and mitosis (phospho-H3) in edge cells. (*a*) PKC*ζ* shows apical localization in non-edge cells but is absent from the edge cells. Supracellular cables perpendicular to the migration direction are seen at the border of the edge cells (arrowhead). Arrow, no localization at the leading edge of the leading cell. (*b,c*) Both RHOA (*b*) and RAC1 (*c*) show expression in the cytoplasm of the edge cells. Strong localization is seen in the ventral-facing irregular shaped protrusions of the edge cells (arrow), also seen in some non-edge cells (arrowhead in (*c*)). (*d,e*) Both E-cadherin and CTNNB are strongly expressed at the junctions between non-edge cells, but both are absent from the leading front of the edge cells (arrow). Supracellular cables perpendicular to the migration direction are seen at the border of the edge cells (arrowhead) (*f*) No phospho-H3-positive cells are observed in the edge cell regions indicating no proliferating cells. The bottom images under each panel show a slice-view from the confocal image stacks. Left-to-right: proximal-to-distal. Blue colour: DAPI stained nuclei. Brackets: edge cells. Scale bar: 100 µm.

**Figure 4.**
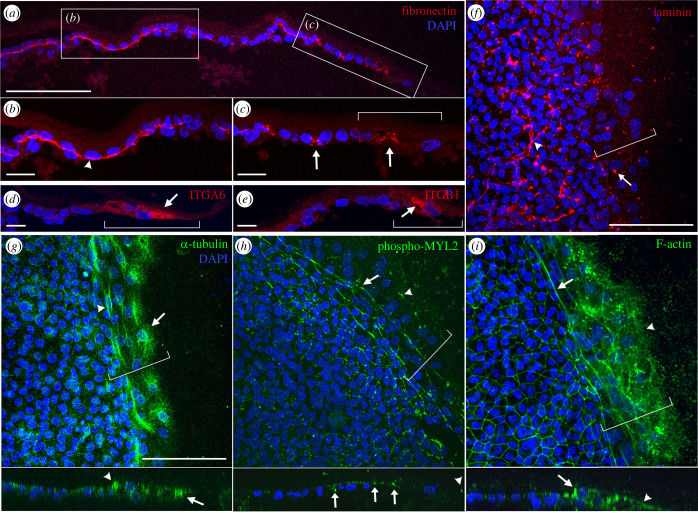
The expression of markers of cell-to-ECM adhesion (fibronectin, ITGA6, ITGB1 and laminin) and cytoskeleton (α-tubulin, phospho-MYL2 and F-actin) in edge cells. (*a*) Fibronectin shows basal localization under the non-edge cells (arrowhead, (*b*)), while disconnected and sparsely distributed expression is observed in the edge cells (arrows, (*c*)). (*d*,*e*) ITGA6 (*d*) and ITGB1 (*e*) are specifically expressed in the leading zone (arrows). (*f*) Laminin shows punctate expression in the edge regions (arrow). (*g*) α-tubulin exhibits mesh-like localization in the lamellipodia of the leading zone (arrow). Supracellular cables (2–3 cells thick) perpendicular to the migration direction are seen at the border of the edge cells (arrowhead). (*h*,*i*) Phospho-MYL2 (*h*) and F-actin show two to three layers of supracellular cables (actomyosin cables) perpendicular to the direction of migration (arrows in (*h,i*)). Phospho-MYL2 also exhibits punctate expression at the tip of the leading zone (arrowhead, (*h*)). F-actin is also expressed in the cytoplasm of the edge cells (arrowhead, (*i*)). The images under (*g–i*) show a slice-view from the confocal image stacks. Left-to-right: proximal-to-distal. Blue colour: DAPI stained nuclei. Brackets: edge cells. Scale bars: 100 µm (*b–e*) and 20 µm in all others.

Cells that migrate collectively have unique cellular properties: the cells in the leading zone exhibit filopodial and lamellipodial activity at their leading face while keeping adhesion at their lateral and rear faces to cells that follow them, which in some cases defines a distinct structure comprising a group of cells that migrate collectively as a unit, which has been considered as a ‘giant supracell’ [15,16]. To investigate this in chick edge cells, immunohistochemistry was conducted to visualize the spatial distribution of a variety of cellular components (figures 3 and 4).

First, we checked the localization of cell polarity markers in the edge cells. PKC*ζ* is localized apically in epithelialized cells in the early chick embryo [13]. In non-edge cells of the area opaca, it is also localized apically, but is not detectable in the edge cells (figure 3*a*), suggesting that the latter lack apical-basal polarity. RHOA and RAC1, members of the Rho family of small GTPases that regulate actin dynamics, show localized expression in migrating cells [17,18]. In the edge cells, both of them show strong localization in the ventral-facing protrusions and elsewhere in the cytoplasm (figure 3*b,c*). Similar protrusions can be seen in a subset of non-edge cells in the outer area opaca (figure 3*b,c*). E-cadherin and CTNNB are components of adherens junctions [19]; they are absent from the leading face of the edge cells but strongly expressed at junctions between non-edge cells (figure 3*d,e*), as previously shown [7] Notably, in the case of PKC*ζ*, E-cadherin and CTNNB, supracellular cables perpendicular to the direction of migration can be seen at the border between edge cells and non-edge cells (figure 3*a,b,d,e*). Perhaps surprisingly given the rapid expansion of the embryo, we could observe no phospho-histone H3-positive cells in the edge cells indicating that the edge cells may not be proliferating; by contrast, there are many positive cells in non-edge regions (figure 3*f*). This is consistent with a previous study that showed that edge cells do not incorporate BrdU [7].

Next, we investigated markers of the ECM and for cellular components that participate in cell-substrate adhesion (figure 4*a–f*). Fibronectin, normally expressed basally in epithelial cells as a continuous sheet [20], is expressed as a sheet under non-edge cells (figure 4*a,b*). However, in sections through edge cells, staining is sparse, consistent with organization as cables oriented parallel to the direction of migration [21] (figure 4*a,c*). Another ECM molecule, laminin, shows punctate staining in edge cells (figure 4*f*) as previously reported [7]. The ECM receptors ITGA6 and ITGB1 are strongly and specifically localized to the edge cells (figure 4*d,e*). As most migrating cells exhibit unique cytoskeletal dynamics [15], we investigated markers of some of these. α-tubulin staining reveals a mesh-like structure in the lamellipodia of the leading-edge cells, while supracellular cables (2–3 cells thick) perpendicular to the migration direction can be observed at the border between edge cells and non-edge cells (figure 4*g*). Phospho-MYL2 and F-actin (phalloidin) staining reveals 2–3 layers of supracellular cables (actomyosin cables) in the edge cells (figure 4*h,i*), suggesting tension perpendicular to the direction of migration [15]. Punctate localization of phospho-MYL2 was also observed at the free margin of the leading-edge cells (arrowhead, figure 4*h*). The expression of F-actin is strong in the edge cells, mostly in the cytoplasm (figure 4*i*). Together, these immunohistochemistry data demonstrate unique molecular features of the edge cells compared to neighbouring non-edge cells.

### 2.4. Live imaging of edge cells during epiboly

**Figure 5.**
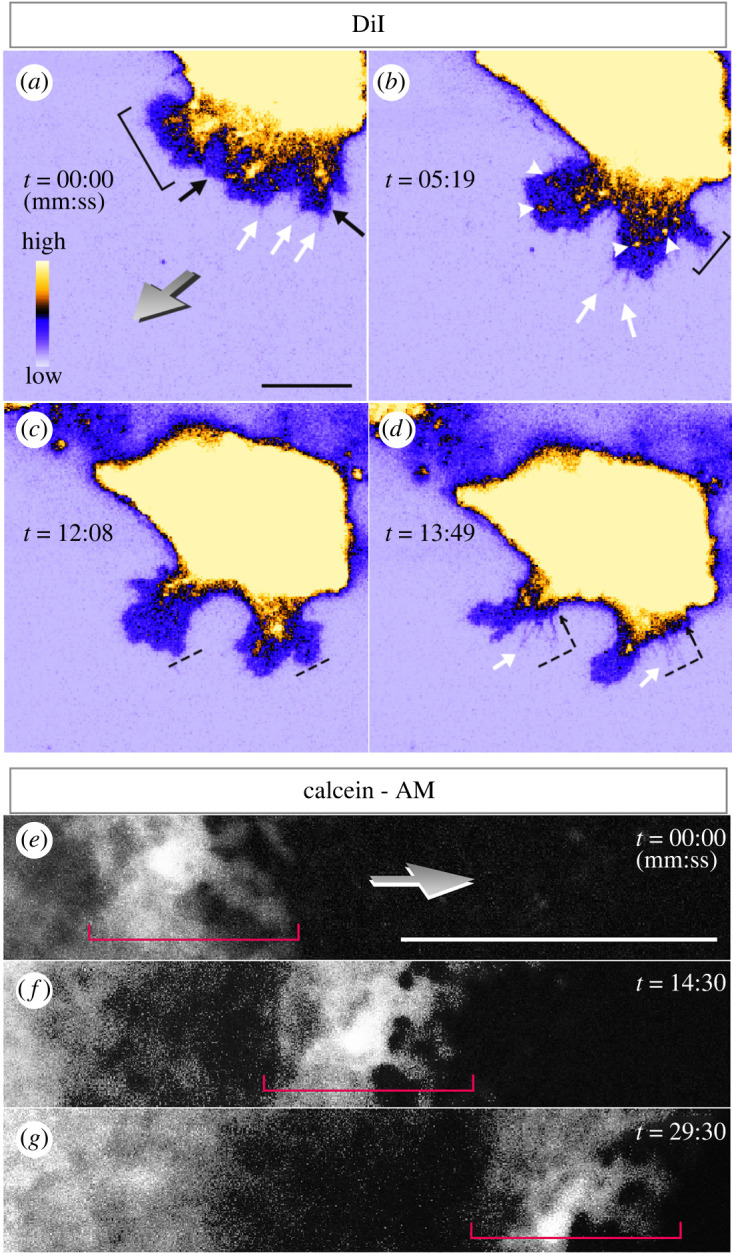
Dynamics of edge cell migration using different staining methods. Frames from a time-lapse sequence showing membrane activity of edge cells during migration using DiI staining (*a–d*) and Calcein-AM staining (*e–g*). (*a*–*d*): DiI staining). Retraction (bracket in (*a*)) and de novo generation (bracket in (*b*)) of lamellipodia are observed. Simultaneously, many highly active filopodia (white arrows) are observed protruding beyond the edge of the lamellipodia. In the lamellipodia, rapid appearance and disappearance of fluorescent localization (arrowheads) is observed, which may correspond to focal adhesions. During cell crawling (dashes in (*c*) and dashed- arrows in (*d*), the lamellipodia retract while the filopodia keep their attachments (*c*,*d*). The pseudo-colour scale reflects fluorescence intensity. Black arrows: lamellipodia. (*e–g*) Calcein-AM staining. Active lamellipodial activity of the membrane of edge cells is observed. The staining clearly marks the extent of the edge region (red brackets). Grey arrow in (*a*,*e*): direction of edge cell migration. Scale bar: 20 µm in (*a*), 100 µm in (*e*).

To study the migratory behaviour of edge cells, we used two different approaches to observe different aspects of edge cell behaviour. First, we immersed the entire embryo in a lipophilic dye, CM-DiI (CellTracker) to label all cell membranes; this revealed details of lamellipodial and filopodial actions at the membranes of edge cells (figure 5*a–d*; electronic supplementary material, movie S1). Lamellipodia were constantly generated and resorbed at the migratory front of the outermost edge cell (figure 5*a–d*; electronic supplementary material, movie S1). Numerous very thin filopodia grew out from the periphery of the lamellipodia (figure 5*a,b,d*; electronic supplementary material, movie S1), as previously reported [10]. Rapid changes in local fluorescence intensity were observed in these lamellipodia, resembling the focal adhesion points of migrating cells [22,23] (figure 5*b*; electronic supplementary material, movie S1). As the edge cell moves forward, the lamellipodia retract away from the direction of migration, while filopodia keep their attachment to the VM and do not retract. This suggests a possible combinatorial action of the lamellipodia and filopodia of the edge cells for their migration, as shown in a model of rapid wound healing [24]. Next, we stained the embryo with Calcein-AM (figure 5*e–g*). This revealed details of lamellipodial action and clearly marks the width of the edge region (red brackets in figure 5*e–g*; electronic supplementary material, movie S2). Our data confirm that very dynamic changes in morphology take place at the outermost front of the edge cells during chick embryo expansion.

In conclusion, we describe some cellular and molecular characteristics of the edge region, specialized for blastoderm expansion on the VM. This reveals that the region is made up of at least four molecularly and anatomically distinct zones. The molecular markers hint at possible roles of WNT signalling. We propose that the edge region could be a useful model to study other general processes, such as wound healing and cancer cell metastasis, which appear to involve similar migratory behaviours.

## 3. Materials and methods

### 3.1. Embryo harvest, fixation and whole-mount *in situ* hybridization

Fertilized White Leghorn hens' eggs were obtained from Henry Stewart, UK, and incubated for 12 h or 24 h to obtain embryos at HH stage 2–3 or 6–7 [14], respectively, or 3 days (about HH stage 19) at 38°C. To keep the VM attached to the embryo, fixation was carried out as a series of sequential steps during embryo harvest. After opening the egg shell, the embryo was brought to the top by rolling the yolk with a spatula. Two to three drops of 4% paraformaldehyde (PFA) in calcium-magnesium free phosphate-buffered saline (PBS) (pH7.4) were applied to cover the embryo and the adjacent VM. After 5–10 s, an area of VM slightly larger than the embryo was cut and lifted out with a spoon-spatula. Two to three drops of 4% PFA were applied directly to the embryo on the spatula for another 5–10 s. Embryos still attached to their membrane were cleared of adherent yolk while submerged in Pannett-Compton saline [25] using streams of saline from a glass Pasteur pipette. They were then fixed in 4% PFA either at 4°C overnight prior to whole-mount *in situ* hybridization, or at room temperature for 1 h prior to immunohistochemistry. Whole-mount *in situ* hybridization was performed as previously described [26,27]. The probes used were *RNH1* (ChEST73n20), *SNAI2* [28], *DACT2* [29], *MSX1* [30], *TGM4* (ChEST698a23), *GATA2* [31], *LHX1* (ChEST389n6) and *DKK1* [32]. For double *in situ* hybridization, the first probe was labelled with fluorescein isothiocyanate and cyan colour development was done with 5-bromo-4-chloro-3-indolyl phosphate (BCIP) only. The second probe was labelled with digoxigenin and dark purple colour development was done with nitro blue tetrazolium plus BCIP. After imaging as whole mounts, embryos were embedded in paraffin and sectioned at 10 µm in a Zeiss Microm microtome. Sections were mounted with Vectashield Plus antifade mounting medium which contains 4′,6-diamidino-2-phenylindole (DAPI) (H-2000, Vector Laboratories) as a nuclear stain. Photographs of sections and whole mounts were obtained with an Olympus Vanox-T microscope with a QImaging Retiga 2000R camera.

### 3.2. Live imaging

Two different dyes were used for live staining of embryos and imaging; CM-DiI (C7001, Invitrogen) and Calcein-AM (17783, Sigma-Aldrich). For CM-DiI staining, embryos were incubated in PBS containing 4 µM CM-DiI in 0.2% dimethyl formamide in Pannett-Compton saline at 38°C for 30 min and then washed with PBS three times. The embryo was then cultured using a modification of the New culture method [33,34] at 38°C for at least 1 h. For Calcein-AM staining, the embryos were first set-up for culture, and then a few drops of 10 µM Calcein-AM in PBS were applied to the edge region of the embryos. The embryo was then imaged with a Leica SPE inverted confocal microscope at 38°C.

### 3.3. Immunohistochemistry and antibodies

For whole-mount immunostaining, fixed embryos were dehydrated with ice-cold methanol and then rehydrated gradually in steps by adding PBS containing 1% Triton X-100 (PBST). After further washing three times for 15 min with PBST, they were blocked with blocking buffer (PBST containing 5% normal goat serum and 0.02% thimerosal) for 2–6 h at room temperature on a rocker. The embryos were then incubated at 4°C for 2–3 days with primary antibodies: PKC*ζ* (SC-216, Santa Cruz), RHOA (SC-179, Santa Cruz), RAC1 (05-389, Millipore), E-cadherin (610182, BD Biosciences), β-catenin (C7207, Sigma), phospho-H3 (06-570, Millipore), laminin (31 or 31-2, Developmental Studies Hybridoma Bank, DSHB), α-tubulin (2125S, Cell Signaling Technology) and phospho-MYL2 (3674S, Cell Signaling Technology), all diluted 1 : 400 except the DSHB antibodies which were diluted 1 : 10. After washing three times with PBST, the embryos were incubated at 4°C for 1 day with fluorescently labelled secondary antibodies: Alexa Fluor 488-conjugated goat anti-mouse IgG (A21202, Invitrogen), Alexa Fluor 488-conjugated goat anti-rabbit IgG (A11008, Invitrogen) or Cy3-conjugated anti-mouse IgG (115-165-073, Jackson), all diluted 1 : 200. For filamentous actin staining, embryos were incubated with rhodamine-phalloidin (Invitrogen R415). For fibronectin staining (VA1, DSHB), integrin-α6 (P2C62C4, DSHB) and integrin-β1 (V2E9, DSHB), fixed embryos were embedded in paraffin and sectioned, then antigen retrieval was conducted in 10 mM sodium citrate (pH 6) for 10 min at 98°C. Then, sectioned tissues were incubated for 1 day at 4°C with primary antibodies, and for 1 h at room temperature with secondary antibodies. For nuclear staining, 2.5 µg ml^−1^ of DAPI was applied to the embryos or sections for 10 min and then washed thoroughly. For whole embryo mounting, the embryo was transferred onto a slide dorsal side-up and flattened carefully, then the excessive solution was removed using a paper tissue. Twenty microlitres of mounting medium (H-1000-10, VECTASHIELD) were applied before covering with a coverslip. The stained embryos and sectioned tissues were imaged with a Leica SPE1 confocal microscope. The images were processed using Fiji software [35] to obtain a maximum projection or a slice view.

## Data Availability

The data are provided in the electronic supplementary material [36].
